# Prevalence and factors associated with underweight among 15–49-year-old women in Sierra Leone: a secondary data analysis of Sierra Leone demographic health survey of 2019

**DOI:** 10.1186/s12905-023-02358-4

**Published:** 2023-04-22

**Authors:** Eric Nzirakaindi Ikoona, Mame Awa Toure, Amon Njenga, Lucy Namulemo, Ronald Kaluya, Kassim Kamara, Freddy Wathum Drinkwater Oyat, Judith Aloyo, David Lagoro Kitara

**Affiliations:** 1 ICAP at Columbia University, Freetown, Sierra Leone; 2 Foothills Community Based Interventions, Monticello, KY USA; 3 Lindsey Wilson College, School of Professional Counseling, Kentucky, USA; 4 Uganda Counseling and Support Services, Kampala, Uganda; 5 Directorate of Health Security and Emergencies, Ministry of Health and Sanitation, Free Town, Sierra Leone; 6 Uganda Medical Association (UMA), UMA-Acholi Branch, Gulu City, Uganda; 7 Rhites-N, Acholi, Gulu City, Uganda; 8 Department of Surgery, Harvard University and Faculty at Gulu University, Faculty of Medicine, P.0. Box 166, Gulu City, Uganda

**Keywords:** Underweight, Women, Reproductive age (15–49 years), Sierra Leone, Undernutrition, DHS-2019

## Abstract

**Background:**

Women are at higher risks of being underweight than men due to biological, socio-economic, and cultural factors. Underweight women have high risks of poor obstetric outcomes. We aimed to determine the prevalence and factors associated with being underweight among women of reproductive age (15–49 years) in Sierra Leone.

**Methods:**

We used Sierra Leone Demographic and Health Survey (2019-SLDHS) data of 7,514 women aged 15 to 49 years, excluding pregnant, post-natal, lactating, and post-menopausal women. A multistage stratified sampling approach was used to select study participants, and data was collected using validated questionnaires. A multivariable logistic regression analysis was used to determine factors associated with underweight among 15–49-year-old women in Sierra Leone. Ethical approval for the study was obtained.

**Results:**

The prevalence of underweight was 6.7% (502/7,514). Underweight was likely among age-group of 15–24 years, AOR = 2.50,95%CI:2.39–2.60;*p* < 0.001 compared to 25–34 year age-group and likely among women with parity of one to four, AOR = 1.48,95%CI:1.08–2.03;*p* = 0.015 compared to women who never gave birth. Underweight was unlikely among women who did not listen to radios AOR = 0.67,95%CI:0.55–0.83;*p* < 0.001 compared to those who did; women from the north AOR = 0.73,95%CI:0.56–0.96;*p* = 0.026 compared to the east, and not married women AOR = 0.59,95%CI:0.47–0.76;*p* < 0.001 compared to married. All household wealth indices were not significantly associated with underweight.

**Conclusion:**

The prevalence of underweight among women in the reproductive age (15–49 years) in Sierra Leone was 6.7% and it is lower compared to global and most sub-Saharan African data. Factors associated with underweight were 15–24-year age-group, and parity of one to four. Being underweight was unlikely among women who did not listen to radios, women from the north and not married. All household wealth indices were not significantly associated with underweight. Even though household wealth indices were not significantly associated with being underweight, most underweight women 68.7% (345/502) were in the poorest, poorer, and middle household wealth indices. The need to address socio-economic determinants of underweight among women (aged 15–49 years) due to household poverty is a priority in Sierra Leone.

## Background

According to the 2014 global estimates, being underweight affects around 462 million adults, representing a severe problem among reproductive-age women for their health, and nutrition of their off springs [1]. Low pregestational Body Mass Index (BMI) among women in the reproductive age is an essential determinant of adverse newborn and child outcomes, such as preterm births, low birth weights, under-five mortalities, poor mental and physical developments [2, 3]. In the 2018 Global Nutrition Report, undernutrition has slightly declined over the years, but anemia has risen to 32.8% among women [4].

According to the 2017 United Nations International Children's Emergency Fund (UNICEF) led state of food security and nutrition report, global undernutrition prevalence has decreased since the early 2000s [5]. However, the decline in undernutrition has been less than 20% globally, and has begun to reverse since 2015 [5, 6]. Because of continuous global increase in the prevalence of overweight, it’s prevalence now exceeds underweight in all regions of the world [5–7].

The ineffective tackling of underweight problems combined with the encroaching problem of overweight has left many low-and-middle-income nations under the weight of a double burden of malnutrition (DBM) [6, 7]. It is important to note that maternal and child nutrition are good indicators of a society's overall wellbeing [8]. As observed worldwide, about 10% of women aged 20 to 49 years are underweight [9], with most significant burdens observed in low-income countries [10]. In addition, underweight is considered an indicator of undernutrition in an adult with no underlying comorbidities and it is defined as a body mass index (BMI) less than 18.5 kg/m^2^ [11, 12].

In low-income countries, women are at a high risk of unmet nutrient requirements because of inadequate food supply, mainly attributed to financial constraints [12]. Women have a higher risk of undernutrition than men due to biological, cultural, and socio-economic factors [9, 13, 14]. Harmful gender norms that favor men over women, such as men being served food first, women eating leftovers [15], and women not inheriting property, are common in developing countries [16, 17]. These norms lead to women having a lower socio-economic status than men [18] and being disproportionately affected by undernutrition [19–21].

Undernutrition has far-reaching consequences in women of reproductive age [22] and are experienced at individual, community, and national levels [23]. At individual level, maternal undernutrition is associated with poor obstetric outcomes, for example increased risks of maternal mortality and morbidity, preterm births, low birth weights, stillbirths, and increased risk of neonatal mortality [24].

In addition, undernutrition reduces economic productivity through reduced labor productivity, high treatment costs, reduced wages, and human capital losses [25–27]. This observation negatively affects communities and national development through reduced family incomes and gross domestic products [25, 27].

Furthermore, undernourished women are more likely to give birth to newborns with low birth weights who are at higher risk of developing malnutrition, hence leading to an inter-generational cycle of malnutrition [28]. Therefore, improving women's nutrition is one way of reducing undernutrition in children [10], and a strong pillar in global efforts to reduce maternal mortality [29].

Overall, findings from this study in Sierra Leone could help inform policymakers to design mitigation strategies to curb underweight prevalence among women of reproductive age (15–49 years).

This study aimed to determine the prevalence and factors associated with underweight among women in the reproductive age of 15 to 49 years in Sierra Leone.

## Methods

### Study design

We conducted a secondary data analysis of the 2019 Sierra Leone Demographic Health Survey (SLDHS) datasets [30].

### Study setting

As of July 2019, Sierra Leone had a population of 8.2 million people in a total land area of 78,000 km^2^ with 23.8% of the population residing in urban areas [31]. Sierra Leone's health system has six levels ranging from the highest level at national referral hospital to the lowest at community level [32]. Agriculture contributes about 24% of Gross Domestic Products (GDP), providing half of the export earnings, and it is the main source of income for 84% of Sierra Leoneans living in rural areas [33].

### Study sampling procedures

**Fig. 1:**
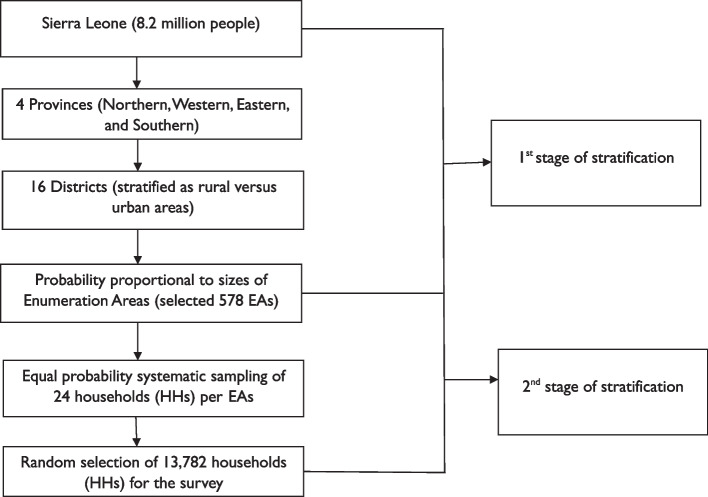
Sampling of participants in the 2019 SLDHS

The 2015 population and housing census of the Republic of Sierra Leone directed by Statistics Sierra Leone (Stats SL) provided the ready-made sampling frame for the 2019 SLDHS survey [30, 34] (Fig. 1). Sierra Leone is administratively divided into four provinces (Eastern, northern, northwestern, and southern plus western areas) which are further subdivided into sixteen districts (Kambia, Port Loko, Bombali, Tonkolili, Moyamba, Bonthe, Bo, Pujehun, Kenema, Kailahum, Kono. Koinadugu, Falaba, Karene, western rural and urban areas) [31–33]. Each district is subdivided into chiefdoms or census wards, and each chiefdom/census ward into sections [31–33]. The 2015 population and housing census of Sierra Leone subdivided each section into convenient census enumeration areas (EAs) [33, 34]. EAs were used as primary sampling units (PSUs) and clusters for the 2019 SLDHS survey [30–34].

The 2019 SLDHS employed a two-stage stratified sampling design and the stratification was achieved by categorizing each district into urban and rural areas (Fig. 1). The list of EAs from the 2015 population and housing census was used for estimating the number of households in a census enumeration area which was, stratified as urban or rural for the 2019 SLDHS sampling frame [30, 31, 33].

In total, thirty-one sampling strata were created in the 2019 SLDHS survey [30]. Samples were selected independently in each stratum via a two-stage selection process [30]. Implicit stratifications were achieved at each lower administrative level by sorting the sampling frame before sample selection according to administrative order and using a probability proportional-to-size selection during the first sampling stage [30]. Accordingly, 578 EAs were selected with a probability proportional to EA size in the first stage of the selection [30]. The size of each enumeration area (EA) was defined by the number of households residing within it.

In the second stage's selection, a fixed number of twenty-four households were selected in every EA through equal probability systematic sampling, resulting in a total sample size of approximately 13,872 households selected and distributed in 578 EAs [30] (Fig. 1).

Household listing was carried out using tablets, and random selection of households to participate in the study was made using computer programming in the selected EAs. The resulting households’ list became the households that were used for the survey in the second stage of the selection process [30].

The survey interviewers talked only to pre-selected households and no replacements or changes of pre-selected households were allowed in the implementation stages of the study to prevent selection bias. Due to non-proportional allocation of samples to the different districts and the possible differences in response rates, sample weights were calculated, added to the data file, and applied so that the results would be representative at national and domain levels [30]. Because the 2019 SLDHS sample was a two-stage stratified cluster sampling, samples selected from the sampling frame and sample weights were calculated separately at each sampling stage and cluster based on sampling probabilities [30].

In addition, the 2019 SLDHS interviewed all women aged 15–49 years in the sampled households who were either permanent residents or visitors who stayed in the households overnight prior to the survey [30]. The man's questionnaire survey was conducted in one-half of the sampled households, and all men aged 15–59 years in the households were included. Additionally, one eligible woman in this subsampled household was randomly selected to answer questions on domestic violence [30]. Similarly, biomarker information was collected only in households selected for the man's survey [30].

### Data collection

The SLDHS data was collected from 14^th^ May 2019 to 31^st^ August 2019 [30]. It was a nationally representative survey carried out by the Bureau of Statistics of Sierra Leone as part of the international MEASURE Demographic Health Surveys (DHS) with support from ICF International and the United States Agency for International Development (USAID) [30]. SLDHS is a periodical demographic health survey conducted every five years in Sierra Leone, and the 2019 was the third survey, with the first in 2010, and the second in 2014 [30].

Five questionnaires were used for the 2019 SLDHS data collection: The Household Questionnaire (HQ), the Woman’s Questionnaire (WQ), the Man’s Questionnaire (MQ), the Biomarker Questionnaire (BQ), and the Fieldworker Questionnaire (FQ). The five questionnaires were based on the DHS Program’s standard Demographic and Health Survey (DHS-7) protocol which were adapted to reflect Sierra Leone’s population and relevant health issues.

Comments on the questionnaires were obtained from various stakeholders representing government ministries and agencies, nongovernmental organizations, and international donors [30]. All five questionnaires were finalized in English, and the 2019 SLDHS used computer-assisted personal interviewing (CAPI) for data collection [30].

The household questionnaire contained the identification of respondents, usual members and visitors in the selected households, background information on each person listed, such as relation to the head of household, age, sex, characteristics of the household’s dwelling unit such as the source of water, type of toilet facilities, type of fuel use for cooking, number of rooms, ownership of livestock, possession of durable goods, mosquito nets and main materials used for the floor, roof and walls of the dwelling place [34].

The woman’s questionnaire contained the identification of respondents, background characteristics (including age, level of education, household size, marital status, residency (rural versus urban), region, parity, assets, lifestyles, work status, sex of household head, and media exposure), birth history and child mortality, knowledge, use and sources of family planning methods, antenatal, delivery, and postnatal care, vaccinations and childhood illnesses, breastfeeding and infant feeding practices, minimum dietary diversity, marriage and sexual activity, fertility preferences (including desire for more children and ideal number of children), women’s work and husband’s background characteristics, knowledge, awareness, and behavior regarding HIV and AIDS, and other sexually transmitted infections (STIs), knowledge, attitudes and behavior related to other health issues (e.g. smoking, watching TV, reading magazines, listening to radios, and alcohol use), female genital cutting, adult and maternal mortality and domestic violence [34].

The man’s questionnaire contained the identification of respondents, background information, reproduction, contraception, marriage and sexual activity, fertility preferences, employment and gender roles, HIV and AIDS and other health issues [34].

The biomarker questionnaire contained the identification of respondents, weight, height, and hemoglobin measurement for children aged, 0–5 years, weight, height, HIV testing and hemoglobin measurement for women aged 15–49 years [34].

The fieldworker questionnaire contained background information on each field worker [34].

On anthropometric measurements, weight was recorded in kilograms (kg) to the nearest one decimal point and was measured using an electronic scale (SECA 878) [30]. Participants’ heights were measured using a stadiometer in centimeters (cm) to one decimal point [30]. Using weights (in kilograms) and heights (meters) of women in the reproductive age (15–49 years), the Body Mass Index (BMI) of individual woman was calculated in Kg/m^2^ and classified according to WHO criteria as; underweight, (< 18.5 kg/m^2^), normal weight (18.5–24.9 kg/m^2^), overweight (25.0–29.9 kg/m^2^) and Obese (≥ 30.0 kg/m^2^).

To calculate each household wealth, we used wealth index (WI) as a proxy indicator of household wealth. This composite index is comprised of household key asset ownership variables which were used to calculate each household wealth index from the 2019 SLDHS data. These variables were the characteristics of the household's dwelling unit, such as the source of water, type of toilet facilities, type of fuel used for cooking, number of rooms, ownership of livestock, possessions of durable goods, mosquito nets, and main materials for the floor, roof, and walls of the dwelling place [34]. Using a computer analysis of household composite factors, household wealth index for each study participant was calculated and categorized as poorest, poorer, middle, richer and richest wealth index (Tables 1, 2 and 5).

**Table 1:** Sociodemographic characteristics of women (15–49 years) in 2019 SLDHS of Sierra Leone

Variables	Frequency (*N* = 7,514)	(Percent) %
**Ages (years)**		
15–24	2,916	38.8
25–34	2,176	29.0
35–49	2,422	32.2
**Parity**		
Never gave birth	1,895	25.2
One to four	3,892	51.8
Five and above	1,727	23.0
**Residence**		
Urban	3,092	41.1
Rural	4,422	58.9
**Sex of the head of household**		
Male	5,356	71.3
Female	2,158	28.7
**The household size**		
Less than six	2,995	39.9
Six and above	4,519	60.1
**Work status**		
Not working	2,280	30.3
Working	5,234	69.7
**Marital status**		
Married	4,795	63.8
Not Married	2,719	36.2
**Regions of residence**		
East	1,579	21.0
North	1,822	24.2
Northwest	1,026	13.7
South	1,831	24.4
Western	1,256	16.7
**Level of education**		
No formal education	3,571	47.5
Primary	1,017	13.5
Secondary	2,641	35.2
Higher	285	3.8
**The wealth Index**		
Poorest	1,533	20.4
Poorer	1,428	19.0
Middle	1,531	20.4
Richer	1,634	21.7
Richest	1,388	18.5
**Watching Television**		
Yes	1,889	25.1
No	5,625	74.9
**Listening to radios**		
Yes	3,142	41.8
No	4,372	58.2
**Reading magazine**		
Yes	489	6.5
No	7.025	93.5
**Smoking cigarettes**		
Yes	224	3.0
No	7,290	97.0
**Alcohol use**		
Yes	667	17.8
No	3,081	82.2
**BMI categories (kg/m**^**2**^**)**		
Underweight (< 18.5)	502	6.7
Normal weight (18.5–24.9)	4,974	66.2
Overweight (25.0–29.9)	1,479	19.7
Obese (≥ 30.0)	559	7.4

Table 1 shows that most Sierra Leone women of reproductive age were in the 15–24 year age-group, 2916/7514(38.8%); parity of one-to-four 3892/7514(51.8%); of rural residence, 4422/7514(58.9%); male headed households, 5356/7514(71.3%); household size of six and above, 4519/7514(60.1%); works, 5234/7514(69.7%); married, 4795/7514(63.8%); from the south, 1831/7514(24.4%); had no formal education, 3571/7514(47.5%); richer in the wealth index, 1634/7514(21.7%); had normal BMI, 4,974/7514(66.2%); did not watch Television, 5625/7514(74.9%); did not listen to radios, 4372/7514(58.2%); did not read magazines, 7025/7514(93.5%); did not smoke cigarettes, 7290/7514(97.0%); and did not use alcohol, 3081/3748(82.2%)

**Table 2:** The wealth indices for women (15–49 years) stratified by regions of Sierra Leone in the 2019 SLDHS

Wealth Indices (WI) (n, %)	Poorest (n, %)	Poorer (n, %)	Middle (n, %)	Richer (n, %)	Richest (n, %)	Total (n, %)
**Regions of Sierra Leone**						
Eastern	453(28.4)	348(23.5)	316(21.4)	254(17.6)	208(13.7)	1579(21.0)
Northern	393(24.6)	395(26.7)	362(24.5)	360(24.9)	312(20.6)	1822(24.2)
Northwestern	179(11.2)	196(13.3)	228(15.4)	221(15.3)	202(13.3)	1026(13.7)
Southern	524(32.8)	367(24.8)	308(20.8)	313(21.7)	319(21.0)	1831(24.4)
Western	47(2.9)	173(11.7)	264(17.9)	297(20.6)	475(31.3)	1256((16.7)
**Total**	**1596(21.2)**	**1479(19.7)**	**1478(19.7)**	**1445(19.2)**	**1516(20.2)**	**7514(100.0)**

In Table 2, most women (15–49 years) with the poorest wealth index were from eastern region, 453(28.4%); poorer from northern, 395(26.7%); middle from northern, 362(24.5%); richer from northern region, 360(24.9%); and richest from western region, 475(31.3%)

Fieldwork monitoring was an integral part of the 2019 SLDHS, and several rounds of monitoring were carried out by the Stats SL, MOHS core teams, coordinators from Stats SL, and ICF staff [34]. Monitors were provided with guidelines for overseeing the fieldwork where weekly field check tables were generated from completed interviews sent to the central office to monitor fieldwork progress, and regular feedback to teams in the field [34]. At the end of data collection exercise, a total of 13,793 households were selected for the sample, of which 13,602 were occupied [34]. Of the occupied households, 13,399 were successfully interviewed, yielding a response rate of 99%. In the interviewed households, 16,099 women aged 15–49 years were identified for individual interviews which were completed with 15,574 women, yielding a response rate of 97%. Meanwhile in the subsample of households selected for the male survey, 7,429 men aged 15–59 years were identified, and 7,197 were successfully interviewed, yielding a response rate of 97% [34].

### Ethical approval

The 2019 SLDHS survey protocol was approved by Sierra Leone Ethics and Scientific Review Committee (SLESRC) and the ICF Institutional Review Board.

### Statistical analysis

SPSS analytic software version 24.0 complex samples package was used for this analysis [35]. We used complex samples package to account for the complex survey sampling while sample weighted data was used to account for unequal probability sampling in different strata. Descriptive statistics and multivariable logistic regressions were used for data analysis.

Frequency tables and proportions/percentages to describe categorical variables were performed, while means and standard deviations were used for continuous variables. Initially, each exposure was assessed separately for its association with the outcome variable (underweight) using bivariable logistic regression analysis, and we presented crude odds ratio (COR), at 95% Confidence Interval (CI), and p-values. Independent variables found insignificant in previous studies and those with p-values less than 0.2 were added to the final multivariable logistic regression model [36–38].

In the multivariable logistic regression analyses, two models were constructed, categorizing independent variables into individual woman, household, and community factors. First, we performed a logistic regression model including individual characteristics only (age, level of education, working status, and marital status).

After that, we constructed a final model including individual characteristics adjusted for household and community characteristics (for example, wealth indices, residences, regions, household sizes, parity, and sex of the head of households). The adjusted odds ratios (AOR) at 95% Confidence Intervals (CI) and *p*-values were calculated with a statistical significance level set at *p*-value < 0.05. In addition, sensitivity analysis was conducted with women who were underweight and those with normal BMI after excluding those with BMI above 25.0.

## Results

The sociodemographic and economic characteristics of women in reproductive age (15–49 years) from the Sierra Leone Demographic Health Survey of 2019 (*N* = 7,514) are presented in Table 1.

### Prevalence of malnutrition

The total number of women in the reproductive age (15–49 years) in the 2019 SLDHS was 15,574. The proportion of women with documented BMI results was 48.2% (7,514/15,574), and the proportion without documented BMI results was 51.8%(8,060/15,574). The mean BMIs was 23.8 kg/m^2^ with a standard deviation (SD) of 4.7. The minimum and maximum BMI measures were 12.8 kg/m^2^ and 99.8 kg/m^2^, respectively. In the whole dataset, there were five outlier BMI variables; the first outlier had BMI of 12.8 kg/m^2^, the second, 14.2 kg/m^2^ and the third, fourth, and fifth had BMI of 98.9 kg/m^2^ each. All these outlier BMI values constituted 0.066% of the total study population (0.026% on the left side, and 0.039% on the right side of the normal distribution curve).

The prevalence of underweight (defined as BMI < 18.5 kg/m^2^) among women of reproductive age (15–49 years) in Sierra Leone in the 2019 DHS was 6.7% (502/7,514).

**Fig. 2:**
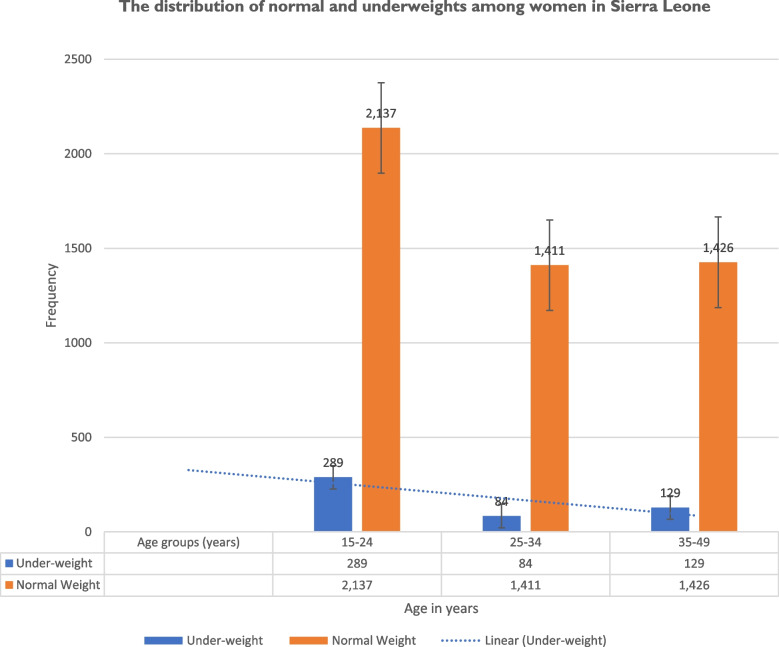
The distribution of normal and underweights in women of reproductive age (15–49 years) in the 2019 SLDHS

The study found that underweight was commonest among 15–24-year age-group, 289/7514(3.8%), followed by 35–49-year age-group, 129/7514(1.7%), and least among 25–34-year age-group, 84/7514(1.1%) (Fig. 2).

The majority of women in reproductive age (15–49 years) in the 2019 SLDHS were in the 15–24 year age-group, 2916/7514(38.8%); parity of one to four, 3892/7514(51.8%); of rural residence, 4422/7514(58.9%); male headed households, 5356/7514(71.3%); household size of six and above, 4519/7514(60.1%); work, 5234/7514(69.7%); married, 4795/7514(63.8%); from the south, 1831/7514(24.4%), had no formal education, 3571/7514(47.5%); in richer wealth index, 1634/7514(21.7%); normal weight (18.5–24.9 kg/m^2^), 4974/7514(66.2%); did not watch TV, 5625/7514(74.9%); did not listen to radios, 4372/7514(58.2%); did not read magazines, 7025/7514(93.5%); did not smoke cigarettes, 7290/7514(97.0%); did not use alcohol, 3081/7541(82.2%) (Table 1).

The household wealth indices for women (15–49 years) stratified by regions of Sierra Leone were as follows; most women in the poorest wealth index were from eastern region, 453(28.4%); poorer from northern, 395(26.7%); middle from northern, 362(24.5%); richer from northern, 360(24.9%); and richest from western region, 475(31.3%) (Table 2).

Also, most participants without formal education were from rural areas 2701/3571(75.6%) compared to 870/3571(24.4%) from urban areas. On the other hand, most educated women were from urban areas 2222/3943(56.4%) compared to 1721/3943(43.6%) from rural areas. Most underweight women were from rural areas (those without formal education constituted, 180/340(52.9%) and the educated, 160/340(47.1%)).

For normal weight, most participants without formal education 1939/2399(80.8%) were from rural areas, while the educated women were from urban areas 1358/2575(52.7%) (Table 3).

**Table 3:** Distribution of participant women (15–49 years) by educational status and residences

**Level of education**	**Type of residence (n, %)**		
	**Urban**	**Rural**	**Total**
No formal education	870(24.4)	2701(75.6)	3571(47.5)
Educated	2222(56.4)	1721(43.6)	3943(53.5)
**Total**	**3092 (41.1)**	**4422 (58.9)**	**7514(100.0)**
**Distribution of underweight women by educational status and places of residence**			
**Level of education**	**Type of residence (n, %)**		
	**Urban**	**Rural**	**Total**
No formal education	31(14.7)	180(85.3)	211(42.0)
Educated	131(45.0)	160(55.0)	291(48.0)
**Total**	**162 (32.3)**	**340(67.7)**	**502(100.0)**
**Distribution of normal weight women by educational status and places of residence**			
**Level of education**	**Type of residence (n, %)**		
	**Urban**	**Rural**	**Total**
No formal education	460(19.2)	1,939(80.8)	2399(48.2)
Educated	1,358(52.7)	1,217(47.3)	2575(51.8)
**Total**	**1818 (36.6)**	**3156 (63.4)**	**4974(100.0)**

In Table 3, most participants without formal education were from rural areas 2701/3571(75.6%) compared to 870/3571(24.4%) from urban areas. On the other hand, most educated women were from urban areas 2222/3943(56.4%) compared to 1721/3943(43.6%) from rural areas. Most underweight women were from rural areas (those without formal education constituting 180/340(52.9%) and the educated, 160/340(47.1%))

For normal weight, most participants without formal education 1939/2399(80.8%) were from rural areas, while the educated women were from urban areas 1358/2575(52.7%)

Likewise, most underweight women were from rural areas 340/502(67.7%) compared to 162/502(32.3%) from urban areas and the educated women constituted 160/502(31.9%), and not educated 180/502(34.6%). In addition, most educated underweight women were in the age-group of 15–24 years 231/502(46.0%).

For normal weight, the majority were among the not educated women from rural areas while the educated from urban areas. The majority of educated women 2575/4974(51.8%) were in the age-group of 15–24 years 1708/4974(34.3%) (Table 4).

**Table 4:** Distribution of women (15–49 years) by weight category, residences, and education category

**Weight categories**		**Places of residence**		
	**Education category**	**Urban**	**Rural**	**Total (n, %)**
**Underweight**	No formal education	31(14.7)	180(85.3)	211(42.0)
	Educated	131(45.0)	160(55.0)	291(58.0)
	Total	162(32.3)	340(67.7)	502(100.0)
**Normal weight**	No formal education	460(19.2)	1939(80.8)	2399(48.2)
	Educated	1358(52.7)	1217(47.3)	2575(51.8)
	Total	1818(36.6)	3156(63.4)	4974(100.0)
**Normal and underweight**	No formal education	491(18.8)	2119(81.2)	2610(47.7)
	Educated	1489(52.0)	1377(48.0)	2866(52.3)
	Total	1980(36.2)	3496(63.80)	5476(100.0)
		**Education category**		
	Age-groups	Not educated	Educated	Total
	15–24	58(20.1)	231(79.9)	289(57.6)
	25–34	44(52.4)	40(47.6)	84(16.7)
**Underweight**	35–49	109(84.5)	20(15.5)	129 (26.7)
	Total	211(42.0)	291(58.0)	502(100.0)
		**Education category**		
	Age-groups	Not educated	Educated	Total
	15–24	429(20.1)	1708(79.9)	2137(43.0)
	25–34	779(55.2)	632(44.8)	1411(28.4)
**Normal weight**	35–49	1191(83.5)	235(16.5)	1426(28.6)
	Total	2399(48.2)	2575(51.8)	4974(100.0)

In Table 4, most underweight women were from rural areas 340/502(67.7%) compared to 162/502(32.3%) from urban areas. The educated women constituted 160/502(31.9%), and not educated 180/502(34.6%) of the rural underweight. Most educated underweight women were in the age-group of 15–24 years 231/502(46.0%). For normal weight, the majority were among the not educated women from rural areas while the educated from urban areas. The majority of educated women 2575/4974(51.8%) were in the age-group of 15–24 years 1708/4974(34.3%)

The unadjusted and adjusted Odds ratios for underweight at multivariable logistic regression analyses for women in reproductive age (15–49 years) from the 2019 SLDHS showed that factors associated with being underweight were likely among age-group of 15–24 years, AOR = 2.50,95%CI:2.39–2.60;*p* < 0.001 compared to 25–34 year age-group and parity of one to four, AOR = 1.48,95%CI:1.08–2.03;*p* = 0.015 compared to women who never gave birth. Underweight was unlikely among women who did not listen to radios AOR = 0.67,95%CI:0.55–0.83;*p* < 0.001 compared to those who did; women from the north AOR = 0.73,95%CI:0.56–0.96;*p* = 0.026 compared to the east, and not married women AOR = 0.59,95%CI:0.47–0.76;*p* < 0.001 compared to married. All household wealth indices were not significantly associated with underweight (Table 5).

**Table 5:** Unadjusted and adjusted values of underweight at bivariable and multivariable regression analyses for women (15–49 years) in the 2019 Sierra Leone DHS

Variable	Underweight (*N* = 502) (n, %)	Normal weight *N* = (4,974) n (%)	Unadjusted COR	95% CI	*p* value	Adjusted POR	95% CI	*p* value
**Age (years)**								
15–24	289(11.9)	2,137(88.1)	2.271	(2.069–2.467)	< .001	2.499	(2.394–2.596)	< .001
25–34	84(5.6)	1,411(94.4)	**Reference**			**Reference**		
35–49	129(8.3)	1,426(91.7)	1.519	(1.438–1.632)	< .001	1.679	(1.589–1.694)	.370
**Parity**								
Never gave birth	225(14.5)	1,330(85.5)	**Reference**			**Reference**		
One to four	182(6.7)	2537(93.3)	2.358	(1.918–2.899)	< .001	1.479	(1.079–2.029)	.015
Five and above	95(7.9)	1,107(92.1)	1.971	(1.531–2.538)	< .001	1.362	(0.876–2.117)	.170
**Residence**								
Urban	162(8.2)	1,818(91.8)	**Reference**					
Rural	340(9.7)	3,156(90.3)	0.827	(0.680–1.006)	.057			
**Sex of household head**								
Male	343(8.7)	3,621(91.3)	**Reference**			**Reference**		
Female	159(10.5)	1,353(89.5)	0.806	(0.661–0.983)	.033	0.925	(0.750–1.141)	.469
**Household size**								
Less than six	181(8.4)	1,976(91.6)	**Reference**					
Six and above	321(9.7)	2,998(90.3)	0.855	(0.707–1.035)	.109			
**Work status**								
Not working	191(11.1)	1,529(88.90	**Reference**			**Reference**		
Working	311(8.3)	3,445(91.7)	0.723	(0.598–0.874)	.001	0.944	(0.750–1.189)	.626
**Marital status**								
Married	232(7.0)	3,102(93.0)	**Reference**					
Not Married	270(12.6)	1,872(87.4)	0.519	(0.431–0.624)	< .001	0.594	(0.467–0.755)	< .001
**Region**								
East	96(8.1)	1,082(91.9)	**Reference**			**Reference**		
North	153(10.5)	1,305(89.5)	0.757	(0.579–0.989)	.041	0.734	(0.559–0.963)	.026
Northwest	73(9.2)	724(90.8)	0.88	(0.640–1.210)	.431	0.840	(0.609–1.160)	.290
South	134(10.3)	1,173(89.7)	0.777	(0.590–1.022)	.071	0.776	(0.588–1.204)	.073
Western	46(6.2)	690(93.8)	1.331	(0.925–1.916)	.777	1.385	(0.954–2.011)	.087
**Level of education**								
No educated	211(8.1)	2,399(91.9)	**Reference**					
Primary	96(12.3)	686(87.7)	0.628	(0.487–0.812)	< .001	0.837	(0.624–1.123)	.236
Secondary	185(9.5)	1,755(90.5)	0.834	(0.679–1.026)	.086	1.128	(0.843–1.510)	.417
Higher	10(6.9)	134(93.1)	1.179	(0.611–2.275)	.624	0.764	(0.374–1.562)	.461
**Wealth Index**								
Middle	121(10.3)	1,050(89.7)	**Reference**			**Reference**		
Poorest	104(8.3)	1,156(91.7)	0.781	(0.593–1.028)	.078	0.812	(0.610–1.080)	.153
Poorer	120(10.2)	1,053(89.8)	0.989	(0.757–1.291)	.935	1.077	(0.819–1.415)	.597
Richer	97(9.1)	974(90.9)	0.864	(0.653–1.145)	.309	0.872	(0.645–1.180)	.374
Richest	60(7.5)	741(92.5)	0.703	(0.508–0.971)	.032	0.832	(0.562–1.231)	.357
**Watching TV**								
Yes	98(8.0)	1,123(92.0)	**Reference**					
No	404(9.5)	3,851(90.5)	0.832	(0.661–1.047)	.117			
**Listening to radios**								
Yes	152(7.2)	1,967(92.8)	**Reference**					
No	350(10.4)	3,007(89.6)	0.664	(0.544–0.810)	< .001	0.673	(0.549–0.826)	< .001
**Reading magazines**								
Yes	29(9.5)	276(90.5)	**Reference**					
No	473(9.1)	4,698(90.9)	1.044	(0.704–1.548)	.832			
**Smoking cigarettes**								
Yes	18(11.5)	139(88.5)	**Reference**					
No	484(9.1)	4,835(90.9)	1.294	(0.785–2.132)	.313			
**Alcohol use**								
Yes	35(7.5)	429(92.5)	**Reference**					
No	140(6.7)	2,005(93.3)	1.168	(0.795–1.717)	.428			

*AOR* Adjusted odds ratio, *CI* Confidence interval, *COR* Crude odds ratio, *SLDHS* Sierra Leone demographic and health survey

In Table 5, factors associated with being underweight were likely among age-group of 15–24 years, AOR = 2.50,95%CI:2.39–2.60;*p* < 0.001 compared to 25–34 year age-group and parity of one to four, AOR = 1.48,95%CI:1.08–2.03;p = 0.015 compared to women who never gave birth. Underweight was unlikely among women who did not listen to radios AOR = 0.67,95%CI:0.55–0.83; *p* < 0.001 compared to those who did; women from the north AOR = 0.73,95%CI:0.56–0.96;*p* = 0.026 compared to the east, and not married women AOR = 0.59,95%CI:0.47–0.76;*p* < 0.001 compared to married. All household wealth indices were not significantly associated with underweight

## Discussion

To our knowledge, this study is one of the first to provide evidence on a nationwide prevalence and factors associated with underweight among 15–49-year-old women of reproductive age in Sierra Leone (Tables 1, 2, Figs. 1, 2 and Table 3). To ensure optimum generalizability of our findings, we used a nationally representative data from the Sierra Leone Demographic Health Survey of 2019 (2019-SLDHS) [30] (Fig. 1). Specifically, this study determined the prevalence of underweight among women of reproductive age (15–49 years) in Sierra Leone at 6.7% (502/7,514) (Table 1).

The prevalence of underweight at 6.7% is within a range comparable to many countries in sub-Saharan Africa [39–42]. This prevalence is lower compared to studies conducted in Kenya (9%) [39] and Tanzania (10%) [40] but like that of Nigeria (6.7%) [41]. The underweight prevalence is also within the range of 5 to 20% reported among women (15–49 years) in the African continent [41].

In a study by Senbanjo et al*.,* from one state of Lagos in Nigeria for example [41], only women aged 15–39 years were included in the survey while the other two studies from Tanzania [40] and Kenya [39] included women aged 15–49 years like our current study. In addition, a World Food Program (WFP) study on East African Regional Food Security & Nutrition update found that Uganda has the lowest prevalence of undernutrition in the East African region partly because of better food security among most of its population compared to Djibouti, Somalia, South Sudan, Burundi, and Kenya [42].

Also, equated with Asian countries and globally, this Sierra Leonean’s underweight prevalence is lower compared to Indonesia at 11.2% [4], Bangladesh at 16.5% [43] and globally at 10% [10]. The observed differences in underweight prevalence among women of reproductive age in the five countries (Uganda, Kenya, Tanzania, Nigeria, and Sierra Leone) which are all in sub-Saharan Africa is likely due to differences in characteristics of study participants, country of origin, age-groups, and their food security status.

The odds of being underweight was significant among participants aged 15–24 years and this was two and a half times more likely than those aged 25–34 years (Table 5). This finding is consistent with other studies in India and other sub-Saharan African countries [44–46]. The current finding in Sierra Leone is likely because this age-group (15–24 years) which consists mainly of adolescents experience rapid physical growth, psychosocial, and cognitive development which requires sufficient nutrient intake to cope with the demand of growth and development. This age-group requires an increased need for nutrients which were likely insufficient in the Sierra Leone’s situation [47]. In addition, a similar high underweight prevalence was observed among adolescent girls in south Asia where over 50% of adolescent girls were affected by undernutrition and anemia due to unmet nutrient requirement, inadequate food supply, and intake [48]. Likewise, the prevalence of underweight was reported high among adolescents living in many countries in sub-Saharan Africa, particularly in Ethiopia [49, 50]. This current Sierra Leone’s report on underweight is likely due to household poverty (Table 1) and food insecurity resulting from lack of food available for consumption because diet and dietary habits are the main factors for underweight in adolescents [51–53]. This is supported by a finding in our study that most underweight 345/502(68.7%) women (15–49 years) in this study population, were in households classified in the poorest, poorer, and middle wealth indices (Tables 1 and 2). In addition, most underweight women 289/502(57.6%) were in the 15–24-year age-group (Fig. 2).

Our study also found that most participants 3,571(47.5%) had no formal education (Tables 1, 3 and 4). As most participants hailed from rural areas 4,422(58.9%), we ascertained that the proportion of participants with no formal education was lower among the urban compared to rural participants (Table 4). We found the actual proportion of participants without formal education was at 39.2% in urban compared to 61.1% in rural areas (Tables 3 and 4). We found that most participants in this survey were aged 15–24-years, 2916/7514(38.8%) and this age-group had a higher proportion of formally educated underweight participants at 231/291(79.4%) compared to their older counterparts at 20/291(6.9%) (Tables 1, 3 and 4). The overall number of underweight women without formal education were more in the older age-group (35–49 years) 109/211(51.7%) compared to 20/291(6.9%) among educated participants in the same age-group (Tables 3 and 4).

Even though most underweight women had formal education 291/502(58.0%), its prevalence among women without formal education was lower at 211/502(42.0%). The majority of those who had no formal education were in the age-group of 35–49 years 109/211(51.7%) (Table 4). Lastly, it is important to note that underweight among women (15–49 years) was not significantly associated with the level of education or residence (rural versus urban) of participants in this study population (Tables 3, 4 and 5).

Our study also found that not married women were unlikely of being underweight than married women (Table 5). In contrast to a previous study in Bangladesh in a pooled analysis, it found that not being married was positively associated with underweight [54]. As well, two previous studies in Ethiopia and Iran are inconsistent with our study where not married were more likely of being underweight compared to married women [55, 56].

Many reports from developing countries show that being married provides women with more excellent financial stability, which in turn works as a protective factor from being underweight [57, 58]. Other factors, such as the use of contraceptive pills, and weight gain in the postpartum period, are more likely to be prevalent among married women in many countries’ contexts [57, 58]. One study in Ethiopia showed that women's nutritional status is affected by lactation, family planning method utilization, lack of education, illnesses, and poor dietary habits [59]. Of note, our current study excluded pregnant, post-natal, and postpartum women, perhaps explaining the inconsistent findings of our study compared to other studies from the African continent.

So, the hypothesis that married women get protected from being underweight because of social shields should be explained in the context of countries, regions, and continents. There is a need for proper and factual explanation on the plausible hypothesis on social protection of married women from being underweight. This warrants a deeper exploration of the socio-cultural dynamics of Sierra Leone communities because our current findings are in contrast with trending information and what has been seen in Ethiopia and Iran [55, 56]. As expected, further studies will be required to establish or disprove any plausible causal connections between not married and not being underweight.

Of special interest from our study was that parity of one to four was one and half times more likely of being underweight compared to women who never gave birth (Table 5). This additional information provides important direction for further enquiry, the negative effect of parity of one to four children on underweight among women (15–49 years) in Sierra Leone (Table 5). This finding in Sierra Leone on parity is consistent with studies in Maldives [60], Burundi, and Ethiopia [61], where higher parity of more than two children were negatively associated with underweight among women of reproductive age. Experts suggest that parity as a risk factor of underweight in women of child-bearing age could reflect multiple reproductive cycles within short intervals which does not allow for sufficient replenishment of women body’s nutrient stock [62]. They argue that women are physiologically vulnerable to malnutrition especially with reproductive functions such as pregnancies and breastfeeding often increasing nutritional requirements [62, 63]. Again, it is said that women in poverty-stricken settings where food insecurity is endemic are often engaged in energy demanding agricultural occupations that often leaves them nutritionally depleted [24]. Endemic household food insecurity provides a reasonable explanation for parity of one to four as a risk factor for underweight among Sierra Leone’s women in reproductive age.

Also, our study found that it was unlikely of being underweight among residents of northern Sierra Leone compared to the east although, there was no significant associations between underweight and northwestern, western, and southern regions of Sierra Leone compared to the east (Table 5). Previous studies showed that regions of residence were associated with underweight in similar low-income African settings [39, 64, 65] and Afghanistan [66]. Similar DHS studies in Uganda found a high prevalence of underweight among women (15–49 years) residents of northeastern region of Uganda who are the poorest and most food insecure [67, 68]. Finding in northeastern Uganda was likely because the region suffers frequent prolonged annual droughts and long civil unrests which significantly affect agricultural production and economy compared to other parts of the country without civil conflicts [68]. In this, experts suggest that decreased agricultural production and poor economy in northeastern Uganda was mainly due to prolonged annual droughts and civil war-induced food insecurity [68–70]. Further, it was proposed that reduction in food production coupled with decreased availability and access of food to the population was common in that region [68–70] and leads to inadequate food in quality and quantity, risking the population from being underweight [68].

Too, most population in northeastern region unlike other regions of Uganda are pastoralists/nomads, and this affect their consumption of food crops as they focus mainly on rearing livestock and move from one location to another frequently [68]. Of note, pastoralists/nomads in Ethiopian pastoral communities, like some communities in East African countries have increased risks of being underweight [69].

In this, a previous report from Sierra Leone showed that nearly half a million children under five years suffer from stunting, while 30,000 suffer from malnutrition and were at immediate risk of death due to inadequate dietary intake, poverty, and high burden of diseases [71]. Some experts argue that there are four primary factors contributing to Sierra Leone's overwhelming poverty: corruption, not a well-established educational system, absence of civil right activities, and poor infrastructures [71]. They argue that these four factors make poverty challenging to beat in Sierra Leone as they have become systemic problems [71].

However, we the authors argue that researchers should not under look the uniqueness of the characteristics of the population in northern Sierra Leone [71]. The culture, tribes, social networks, religious practices, marital arrangements, socioeconomic activities, environment, household wealth indices, social dynamics, and family support systems of the population in northern Sierra Leone which are exclusive may in part explain their unlikeliness of being underweight compared to eastern region [71].

We, the authors propose that additional studies are warranted to determine why underweight is unlikely in northern Sierra Leone compared to eastern region as this current finding presents a unique scenario in a country afflicted by similar challenges but have different effects on northern region compared to other regions (Tables 2 and 5).

### Listening to radios

Our study found that not listening to radios was protective against being underweight among women of reproductive age (15–49 years) in Sierra Leone (Table 5). This is in contrast with a study in Botswana which found that approximately 12.9% of women who did not listen to radios the previous week compared to 11.1% who did, had a low BMI or were underweight [72]. Overall, a higher proportion of women who never listened to radios at least once a week had a higher prevalence of underweight compared to those who did [72]. This finding is inconsistent with our current study in Sierra Leone where not listening to radios was protective of being underweight (Table 5). In addition, findings from Botswana show that young adult women who lacked access to mass media were at greater risks of underweight [72].

As previously observed, radios are vital sources of information on various issues such as health communications and promotion [72]. Through radios, people receive and learn messages about healthy eating behaviors and lifestyles [72]. Thus, it was assumed that those who owned radios were expected to be better informed about food, diet, healthy lifestyles and were able to learn and adopt healthier lifestyle [72].

The assumption in the Botswana study was that participants without a radio did not know about healthy eating behaviors and lifestyles or they could not have access to information on healthy eating behaviors from other sources other than radios and were more likely of being underweight [72].

Interestingly, there are other sources of information to women in the reproductive age in African communities other than radios for example, from health workers, midwives, elders, friends, family members, social networks, traditional leaders, older women, mosques, churches, internet, mobile phones, social media, and others that allow women to get information. Whether these additional sources of reproductive health information to women were considered important issues, or ignored, or not included in the options in the study questionnaire will be one of our future areas of enquiry in Sierra Leone.

In addition, many African communities live in villages, gather in village clubs in the evening for socialization, for example, while drinking alcohol whereby news and updates from radios or mobile phones are shared with neighbors but the ownership remains for a person. These extra scenarios that may not have been captured in this study; a self-administered questionnaire using computer-assisted personal interviewing (CAPI) for quantitative data collection attract interests of qualitative researchers to explore more about health information and communications among the study population.

We, the authors posit that the culture, feeding habits, social networks and dynamics, food availability, and economic activities of women in northern Sierra Leone are likely different from Botswana, Uganda, and Ethiopia, and not listening to radios was protective of being underweight.

As observed in our findings, young women (15–24-year age-group) were the most significantly affected by underweight compared to the older age-groups (Table 2, Fig. 2, Tables 3, 4 and 5). For this significant association between underweight and women of 15–24-year age-group, we, the authors propose that introducing school feeding programs in Sierra Leone’s schools is important for mitigating underweight challenges observed among young women in the reproductive age (15–49 years) in schools.

Findings from our study are very important as a special report on Sierra Leone about the status of teenage pregnancy in 2020 shows it is on the rise [67, 73]. MEDICI CON L’AFRICA, CUAMM, Doctors with Africa says that teenage pregnancy is a big problem affecting girls’ and young women’s health, their social, economic, and political empowerment in Sierra Leone [73]. Overall, the report shows that 28% of adolescent girls aged 15–19 years had begun childbearing; 22% have had a live birth, and 6% were pregnant with their first child as of the date of the survey [73]. In addition, a larger proportion of teenagers in rural areas than in urban areas had begun childbearing (34% versus 19%) [73] while at regional level, the proportion of teenagers who had started childbearing was highest in the Southern region (33%) and lowest in the western region (18%) [73]. This report therefore highlights the urgent need for practical interventions to curb underweight among women of reproductive age in Sierra Leone especially among the teenagers and young adolescents.

Overall, our study found that age-group of 15–24-years and parity of one to four were significantly associated with being underweight. Not listening to radios, residents from the northern region and not married were protective factors against underweight among women (15–49 years) in Sierra Leone (Table 5). However, residency (rural versus urban), sex of the head of household, household size, work status, level of education, wealth indices, reading magazines, watching television, smoking cigarettes, and alcohol use were not significant factors of underweight among women of reproductive age (15–49 years) in Sierra Leone (Table 5). Findings from our study in Sierra Leone show a lower prevalence of underweight compared to Indonesia [74] and Ghana [75], even though they are all in low-to-middle-income countries.

### Strengths of this study

This study has many strengths. First, this study utilized a nationally representative sample population of women in the reproductive age (15–49 years) in Sierra Leone. Second, the data quality was assured as the 2019 SLDHS used well-trained field personnels, standardized protocols, and validated tools in data collection processes. In addition, a group of well trained and experienced scientists collected, cleaned, and entered the data with minimal errors in the final dataset. As a result, findings of this study can be generalized to the target population in Sierra Leone and other developing countries. Third, because we used validated tools and calibrated instruments by SLDHS, the generated estimates are more robust than other studies in the context of Sierra Leone. Finally, we used concentration index whose findings are more robust in predicting socio-economic inequalities in a study population.

### Limitations

There are some limitations in this study which warrants further discussions. First, the 2019 SLDHS was a cross-sectional survey conducted among women of reproductive age (15–49 years). As a result, we cannot establish causal associations between explanatory variables and the outcome variable.

Second, due to the absence of some data, several important variables such as food security and dietary diversity were not part of the model in the final analysis. Third, SLDHS did not collect individual household income and expenditures data. The survey used household wealth index as a proxy indicator for household wealth measures which offers limitations to our findings. Fourth, SLDHS collected data only on 15–49-year-old women of reproductive age. However, with the current changes in adolescents' actions and behaviors, there are children less than 15 years who have gone through a full cycle of reproductive health. As a result, we could not ascertain the distribution of underweight among females below 15 years. Finally, most data on predictors of underweight were based on self-reported information and were not verified through record analysis which risks socially acceptable answers, hence social desirability bias.

### Generalizability of results

Results from this study can be generalized to low resource settings in low-and middle-income countries.

## Conclusion

The prevalence of underweight among women in the reproductive age (15–49 years) in Sierra Leone was 6.7% and it is lower compared to global and most sub-Saharan African data. Factors associated with underweight were 15–24-year age-group, and parity of one-to-four. Being underweight was unlikely among women who did not listen to radios, women from the north and not married. All household wealth indices in our study were not significantly associated with being underweight.

Even though household wealth indices were not significantly associated with being underweight, most underweight women 68.7%(345/502) were in the poorest, poorer, and middle household wealth indices. The need to address socio-economic determinants of underweight among women (aged 15–49 years) due to household poverty is a priority in Sierra Leone.

## Data Availability

All datasets supporting this article's conclusion are within this paper and are accessible by a reasonable request to the corresponding author.

## References

[1] World Health Organization (WHO). Malnutrition Fact sheet 2018. http://www.who.int/news-room/fact-sheets/detail/malnutrition. Accessed 15 Oct 2018.

[2] Black RE, Victora CG, Walker SP, Bhutta ZA, Christian P, de Onis M (2013). Maternal and child undernutrition and overweight in low-income and middle-income countries. Lancet.

[3] Reyes Matos U, Mesenburg MA, Victora CG (2020). Socio-economic inequalities in the prevalence of underweight, overweight, and obesity among women aged 20-49 in low- and middle-income countries. Intern J Obesity.

[4] Development Initiatives. Global Nutrition Report: Shining a light to spur action on nutrition. Glob Nutr Rep. 2018;1–164. GLOBALNUTRITIONREPORT.ORG. https://globalnutritionreport.org/documents/352/2018_Global_Nutrition_Report.pdf.

[5] FAO, IFAD, UNICEF, WFP and WHO. The State of Food Security and Nutrition in the World 2018. FAO. 2018. https://www.fao.org/agrifood-economics/publications/detail/en/c/1153252/.

[6] Prentice AM (2018). The double burden of malnutrition in countries passing through the economic transition. Ann Nutr Metab.

[7] Chakraborty PA, Talukder A, Haider SS, Gupta RD (2022). Prevalence, and factors associated with underweight, overweight, and obesity among 15-49-year-old men and women in Timor-Leste. PLoS ONE.

[8] Abbasnezhad MA, Ebrahimzadeh F, Roosta S, Rezapour M, Choghakhori R (2020). Assessment of nutritional status and related factors of lactating women in the urban and rural areas of southwestern Iran: a population-based cross-sectional study. Int J Community Based Nurs Midwifery.

[9] Kshatriya GK, Acharya SK (2016). Gender disparities in the prevalence of undernutrition and the higher risk among young women of Indian tribes. PLoS ONE.

[10] Vir SC (2016). Improving women's nutrition imperative for rapid reduction of childhood stunting in South Asia: coupling of nutrition-specific interventions with nutrition-sensitive measures essential. Matern Child Nutr.

[11] Mtumwa AH, Paul E, Vuai SAH (2016). Determinants of undernutrition among women of reproductive age in Tanzania mainland. South Afr J Clin Nutr.

[12] Sserwanja Q, Mukunya D, Habumugisha T, Mutisya LM, Tuke R, Olal E (2020). Factors associated with undernutrition among 20 to 49-year-old women in Uganda: a secondary analysis of the Uganda demographic health survey 2016. BMC Public Health.

[13] Haseen F (2010). Malnutrition among ultra-poor women in Bangladesh: malnutrition among Bangladeshi women in ultra-poor households: prevalence and determinants.

[14] Clark S, Berrang-Ford L, Lwasa S (2015). The burden and determinants of self-reported acute gastrointestinal illness in an Indigenous Batwa Pygmy population in southwestern Uganda. Epidemiol Infect.

[15] Madjdian DS, Bras HAJ (2016). Family, gender, and Women's nutritional status: a comparison between two Himalayan communities in Nepal. Econ Hist Dev Regions.

[16] Oniang'o R, Mukudi E. "Nutrition and Gender". In Nutrition: A Foundation for Development. Geneva: ACC/SCN. 2002:1–46. https://www.unscn.org/layout/modules/resources/files/Brief1-12EN.pdf.

[17] Björkman-Nyqvist M (2013). Income shocks and gender gaps in education: evidence from Uganda. J Dev Econ.

[18] Ickes SB, Heymsfield GA, Wright TW, Baguma C (2017). Generally, the young mom suffers much: Socio-cultural influences of maternal capabilities and nutrition care in Uganda. Matern Child Nutr.

[19] Vu L, Pulerwitz J, Burnett-Zieman B, Banura C, Okal J, Yam E (2017). Inequitable gender norms from early adolescence to young adulthood in Uganda: tool validation and differences across age groups. J Adolesc Health.

[20] Namy S, Carlson C, Norcini Pala A, Faris D, Knight L, Allen E, Devries K, Naker D (2017). Gender, violence, and resilience among Ugandan adolescents. Child Abuse Negl.

[21] Okot-Okumu J, Oosterveer P. Providing Sanitation for the Urban Poor in Uganda. In: van Vliet B, Spaargaren G, Oosterveer P, editors. Social Perspectives on the Sanitation Challenge. edn. Dordrecht: Springer Netherlands; 2010;49–66.

[22] Melaku YA, Zello GA, Gill TK, Adams RJ, Shi Z (2015). Prevalence, and factors associated with stunting and thinness among adolescent students in northern Ethiopia: a comparison to world health organization standards. Arch Public Health.

[23] Reinhardt K, Fanzo J (2014). Addressing chronic malnutrition through multi-sectoral, sustainable approaches: a review of the causes and consequences. Front Nutr.

[24] Kamal SMM, Hassan CH, Alam GM (2015). Dual burden of underweight and overweight among women in Bangladesh: patterns, prevalence, and sociodemographic correlates. J Health Popul Nutr.

[25] Patterson K, Berrang-Ford L, Lwasa S, Namanya DB, Ford J, Twebaze F (2017). Seasonal variation of food security among the Batwa of Kanungu. Uganda Public Health Nutr.

[26] Hoddinott J, Alderman H, Behrman JR, Haddad L, Horton S (2013). The economic rationale for investing in stunting reduction. Matern Child Nutr.

[27] Desalegn BB, Lambert C, Riedel S, Negese T, Biesalski HK (2018). ethiopian orthodox fasting and lactating mothers: longitudinal study on dietary pattern and nutritional status in rural Tigray, Ethiopia. Int J Environ Res Public Health.

[28] Christian P, Smith ER (2018). Adolescent undernutrition: global burden, physiology, and nutritional risks. Ann Nutr Metab.

[29] Tinker A, Daly P, Green C (1995). Women’s health and nutrition.

[30] Statistics Sierra Leone (Stats SL) and ICF. 2020. Sierra Leone Demographic and Health Survey 2019. Freetown, Sierra Leone, Rockville, Maryland, USA: Stats SL and ICF. 2020. chrome-extension://efaidnbmnnnibpcajpcglclefindmkaj/https://dhsprogram.com/pubs/pdf/FR365/FR365.pdf

[31] Worldometer. The population of Sierra Leone. 2022. https://www.worldometers.info/world-population/sierra-leone-population/

[32] Sierra Leone NHSSP. National Health Sector Strategic Plan 2017 – 2021. chrome-extension://efaidnbmnnnibpcajpcglclefindmkaj/https://extranet.who.int/countryplanningcycles/sites/default/files/planning_cycle_repository/sierra_leone/sierra_leone_nhssp_2017-21_final_sept2017.pdf

[33] World Food Program (WFP). State of Food Security in Sierra Leone 2015 Comprehensive Food Security and Vulnerability Analysis Data collected September - October 2015. 2015 Sierra Leone CFSV. https://efaidnbmnnnibpcajpcglclefindmkaj/https://documents.wfp.org/stellent/groups/public/documents/ena/wfp288316.pdf?iframe.

[34] World Bank (WB). Microdata in Sierra Leone, 2019. Demographic and Health Survey 2019. Microdata Library. 2019. https://microdata.worldbank.org/index.php/catalog/3826.

[35] IBM SPSS Complex Samples. https://www.ibm.com/products/spss-complex-samples.

[36] Lee PH, Burstyn I (2016). Identification of confounder in epidemiologic data contaminated by measurement error in covariates. BMC Med Res Methodol.

[37] Maldonado G, Greenland S (1993). Simulation study of confounder-selection strategies. Am J Epidemiol.

[38] Mickey RM, Greenland S (1989). The impact of confounder selection criteria on effect estimation. Am J Epidemiol.

[39] Kenya National Bureau of Statistics, Ministry of Health/Kenya, National AIDS Control Council/Kenya, Kenya Medical Research Institute, Population NCf, Development/Kenya: Kenya Demographic and Health Survey 2014. Rockville; 2015.

[40] Ministry of Health CD, Gender, Elderly, Children - MoHCDGEC/Tanzania Mainland, Ministry of Health - MoH/Zanzibar, National Bureau of Statistics - NBS/Tanzania, Office of Chief Government Statistician - OCGS/Zanzibar, ICF. Tanzania Demographic and Health Survey and Malaria Indicator Survey (TDHS-MIS), 2015–2016. Dar es Salaam, Tanzania, and Rockville, Maryland, USA: MoHCDGEC, MoH, NBS, OCGS, and ICF; 2016. https://dhsprogram.com/pubs/pdf/fr321/fr321.pdf.

[41] Senbanjo IO, Olayiwola IO, Afolabi WA, et al. Maternal and child under-nutrition in rural and urban communities of Lagos state, Nigeria: the relationship and risk factors. BMC Res Notes. 2013;6(286):1–10. 10.1186/1756-0500-6-286. 10.1186/1756-0500-6-286 PMC3725170 23880121

[42] World Food Program: East Africa Regional Food Security & Nutrition Update (November 2019): https://reliefweb.int/report/ethiopia/east-africa-regional-food-security-nutrition-update-november-2019.

[43] Hashan MR, Gupta RD, Day B, Al Kibria GM (2020). Differences in prevalence and associated factors of underweight and overweight/obesity according to rural-urban residence strata among women of reproductive age in Bangladesh: evidence from a cross- sectional national survey. BMJ Open.

[44] Pengpid S, Peltzer K (2019). Prevalence and correlates of underweight and overweight/obesity among women in India: results from the national family health survey 2015–2016. Diabetes Metab Syndr Obes: Targets Ther.

[45] Al Kibria GM, Swasey K, Hasan MZ, Sharmeen A, Day B (2019). Prevalence and factors associated with underweight, overweight and obesity among women of reproductive age in India. Global Health Res Policy.

[46] Amugsi DA, Dimbuene ZT, Kyobutungi C (2019). Correlates of the double burden of malnutrition among women: an analysis of cross-sectional survey data from sub-Saharan Africa. BMJ Open.

[47] Das Gupta M, Engelman R, Levy J, Gretchen L, Merrick T, Rosen JE (2014). The Power of the 1.8 billion, Adolescents, Youth and the Transformation of the Future, State of World Population, United Nations Population Fund, New York City, NY, USA.

[48] Aguayo VM, Paintal K (2017). Nutrition in adolescent girls in south Asia. BMJ.

[49] Wassiel MM, Gete AA, Yesuf ME, Alene GD, Belay A, Moges T (2015). Predictors of nutritional status of Ethiopian adolescent girls: a community based cross sectional study. BMC Nutrition.

[50] Jaacks LM, Slining MM, Popkin BM (2015). Recent trends in the prevalence of under-and overweight among adolescent girls in low-and-middle-income countries. Pediatr Obes.

[51] Krug I, Villarejo C, Jimenez-Murcia S (2013). Eating-related environmental factors in underweight eating disorders and obesity: are there common vulnerabilities during childhood and early adolescence. Eur Eat Disord Rev.

[52] Noh JW, Kim YE, Park J, Oh IH, Kwon YD (2014). Impact of parental socio-economic status on childhood and adolescent overweight and underweight in Korea. J Epidemiol.

[53] Broussard NH (2012). Food aid and adult nutrition in rural Ethiopia. Agric Econ.

[54] Tanwi TS, Chakrabarty S, Hasanuzzaman S (2019). Double burden of malnutrition among ever-married women in Bangladesh: A pooled analysis. BMC Women's Health.

[55] Abrha S, Shiferaw S, Ahmed KY (2016). Overweight and obesity and its sociodemographic correlate among urban Ethiopian women: Evidence from the 2011 EDHS. BMC Public Health.

[56] Janghorbani M, Amini M, Willett WC, Gouya MM, Delavari A, Alikhani S (2007). First nationwide survey of the prevalence of overweight, underweight, and abdominal obesity in Iranian adults. Obesity.

[57] Begum F, Colman I, McCargar LJ, Bell RC, On behalf of the Alberta Pregnancy Outcomes (2012). Gestational weight gain and early postpartum weight retention in a prospective cohort of alberta women. J Obstet Gynaecol Canada.

[58] Nartea R, Mitoiu BI, Nica AS (2019). Correlation between pregnancy related weight gain, postpartum weight loss and obesity: a prospective study. J Med Life.

[59] Tafa M and Haidar J. Effect of modern family planning use on nutritional status of women of reproductive age group at Tena district, Arsi zone, Oromiya region, Ethiopia: a comparative study. The Ethiopian Journal of Health Development. 2014;28(2). https://ejhd.org/index.php/ejhd/article/view/127.

[60] Hashan MR, Rabbi MF, Haider SS, Das Gupta R (2020). Prevalence and associated factors of underweight, overweight and obesity among women of reproductive age group in the Maldives: Evidence from a nationally representative study. PLOS ONE.

[61] Were JM, Stranges S, Creed IF (2020). Fertility is a key predictor of the double burden of malnutrition among women of child-bearing age in sub-Saharan Africa. J Glob Health.

[62] Lartey A (2008). Maternal and child nutrition in sub-Saharan Africa: Challenges and interventions. Proc Nutr Soc.

[63] Kim SA, Stein AD, Martorell R (2007). Country development and the association between parity and overweight. Int J Obes (Lond).

[64] Mengesha Kassie A, Beletew Abate B, Wudu Kassaw M, Gebremeskel Aragie T. Prevalence of Underweight and Its Associated Factors among Reproductive Age Group Women in Ethiopia: Analysis of the 2016 Ethiopian Demographic and Health Survey Data. J Environ Public Health. 2020;2020:9718714. 10.1155/2020/9718714. 10.1155/2020/9718714 PMC7403906 32802085

[65] Akokuwebe ME, Idemudia ES (2022). Multilevel analysis of urban-rural variations of body weights and individual-level factors among women of childbearing age in Nigeria and South Africa: a cross-sectional survey. Int J Environ Res Public health.

[66] Akseer N, Bhatti Z, Mashal T, Soofi S, Moineddin R, Black RE, Bhutta ZA (2018). Geospatial inequalities and determinants of nutritional status among women and children in Afghanistan: an observational study. Lancet Glob Health.

[67] Wichern J, van Wijk MT, Descheemaeker K, Frelat R, van Asten PJA, Giller KE (2017). Food availability and livelihood strategies among rural households across Uganda. Food Secur.

[68] Turi KN, Christoph MJ, Grigsby-Toussaint DS (2013). Distribution of underweight, overweight and obesity among women and children: results from the 2011 Uganda demographic and health survey. Int J Environ Res Public Health.

[69] Girma W, Genebo T. Determinants of nutritional status of women and children in Ethiopia. Calverton: ORC Macro; 2002;1–32. https://dhsprogram.com/pubs/pdf/fa39/02-nutrition.pdf.

[70] Tusiime HA, Renard R, Smets L (2013). Food aid and household food security in a conflict situation: empirical evidence from northern Uganda. Food Policy.

[71] The World Bank IBRD-IDA. World Health Organization, Global Health Observatory Data Repository/World Health Statistics. Prevalence of anemia among women of reproductive age (% of women ages 15–49) - Sierra Leone. 2000–2019. https://data.worldbank.org/indicator/SH.ANM.ALLW.ZS?locations=SL.

[72] Letamo G, Navaneetham K (2014). Prevalence and determinants of adult under-nutrition in Botswana. PLoS ONE.

[73] MEDICI CON L’AFRICA, CUAMM, Doctors with Africa. Teenage pregnancy in Sierra Leone. https://doctorswithafrica.org/en/wp-content/uploads/sites/2/2020/01/teenage-pregnancy-sierra-leone.pdf.

[74] Pengpid S, Peltzer K (2017). The prevalence of underweight, overweight/obesity and their related lifestyle factors in Indonesia, 2014–15. AIMS Public Heal.

[75] Doku DT, Neupane S (2015). Double burden of malnutrition: increasing overweight and obesity and stall underweight trends among Ghanaian women. BMC Public Health.

